# Assessment of respiratory function in children wearing a N95 mask with or without an exhalation valve: Data compared

**DOI:** 10.1016/j.dib.2021.107550

**Published:** 2021-11-07

**Authors:** Riccardo Lubrano, Silvia Bloise, Alessia Marcellino, Claudia Proietti Ciolli, Alessia Testa, Enrica De Luca, Anna Dilillo, Saverio Mallardo, Sara Isoldi, Vanessa Martucci, Mariateresa Sanseviero, Emanuela Del Giudice, Concetta Malvaso, Claudio Iacovelli, Rita Leone, Donatella Iorfida, Flavia Ventriglia

**Affiliations:** Dipartimento Materno Infantile e di Scienze Urologiche, Sapienza Università di Roma, UOC di Pediatria e Neonatologia - Polo Pontino, Italy

**Keywords:** COVID-19, Facial mask, Respiratory function, Children

## Abstract

In response to the current COVID-19 pandemic, universal face masking represents one of the most important strategies to limit the spread of infection. However, their use in children is still highly debated (Esposito and Principi, 2020; Esposito et al., 2020) and there are few data (Lubrano et al., 2021a, 2021b) describing their possible effects on respiratory function in children.

A dataset in this paper presents a comparison of the data related to the effects on respiratory function of children wearing a filtering facepiece 2 (N95 mask) with or without exhalation valve. 22 healthy children were randomly assigned to two groups, both groups wearing an N95 mask: one without an exhalation valve (group A), another with an exhalation valve (group B).

Children were subjected to a 72 min test: the first 30 min without mask, then 30 min wearing face mask while practiced their usual play activity; finally, 12 min, with face mask in place, while they walked as in a walking test. They were monitored through to microstream capnography system (Rad-97TM with Nomo-Line Capnography, Masimo, Irvine, CA, USA) to log oxygen saturation (SpO2) and respiratory rate (RR).

We use the Wilcoxon test to analyzed the differences between the parameters recorded during the study in group A and B. Data analysis was performed using JMP14.3.0 program for Mac by SAS Institute inc.

**Specifications Table**

**Table:** 

Subject	Public health
Specific subject area	Health education, health promotion, prevention
Type of data	Primary data Tables Figure
How data were acquired	The data were acquired throught to microstream capnography system (Rad-97TM with Nomo-Line Capnography, Masimo, Irvine, CA, USA)
Data format	Raw Analyzed
Parameters for data collection	We involved 22 healthy children at Pediatric Unit of Santa Maria Goretti Hospital, Latina - Sapienza University of Rome. Participants were randomly divided into two groups: Group A was composed of children wearing N95 mask without an exhalation valve, while Group B composed of children wearing N95 mask with an exhalation valve. Children were subjected to a 72 min test: the first 30 min without mask, then 30 min wearing face mask while practiced their usual play activity; finally, 12 min, with face mask in place, while they walked as in a walking test. The parameters for data collection were: oxygen saturation (SpO2), and respiratory rate (RR).
Description of data collection	All Data were recorded during a clinical study conducted at Pediatric Unit of Santa Maria Goretti Hospital, Latina - Sapienza University of Rome. Then alle data were collected on an electronic database and analyzed.
Data source location	Institution: Sapienza Università di Roma, Dipartimento Materno Infantile e di Scienze Urologiche, UOC di Pediatria e Neonatologia - Polo Pontino City: Latina Region: Lazio Country: Italy
Data accessibility	The data are hosted with the article.
Related research article	Riccardo Lubrano, Silvia Bloise, Alessia Marcellino, Claudia Proietti Ciolli, Alessia Testa, Enrica De Luca, Anna Dilillo, Saverio Mallardo, Sara Isoldi, Vanessa Martucci, MariaTeresa Sanseviero, Emanuela Del Giudice, Concetta Malvaso, Claudio Iacovelli, Rita Leone, Donatella Iorfida, Flavia Ventriglia. Effects of N95 Mask Use on Pulmonary Function in Children. J Pediatr. 2021 Oct;237: 143–147. doi: 10.1016/j.jpeds.2021.05.050.

**Value of the Data**

• Despite a mild clinical course in pediatric age ([1], even children can transmit the infection and humoral immunity decays even in this age group [2]. Therefore, any preventive measures should be implemented [3], [4], [5], [6], but empirical evidence is necessary to scientifically address current recommendations [7,8]. These data could clarify some aspects related to possible respiratory effects of the use of mask in children. implementing their use in pediatric age.
• Currently, few data are available in the literature describing the effects on respiratory function with the use of N95 masks by children [9], [10], [11]. Therefore, our results could improve the knowledge of the scientific community about the use of this type of mask in children, promoting their safer and more rational use.
• These findings are useful for other researchers who would like to compare our data with those of other studies on the effects of face masks on respiratory function in pediatric age. In addition, they can be used as a term of comparison for future studies on the use of face masks in children with comorbidities, such as obesity or heart diseases.

## 1 Data Description

These data show existing differences in the effects on respiratory function between children wearing an N95 mask without an exhalation valve (group A), composed by 11 participants, and children wearing an N95 mask with an exhalation valve (group B), composed by 11 participants

At T30, when children not wearing a mask, there were not significative differences in SpO2 and RR between Group A and Group B.

**Fig. 1:**
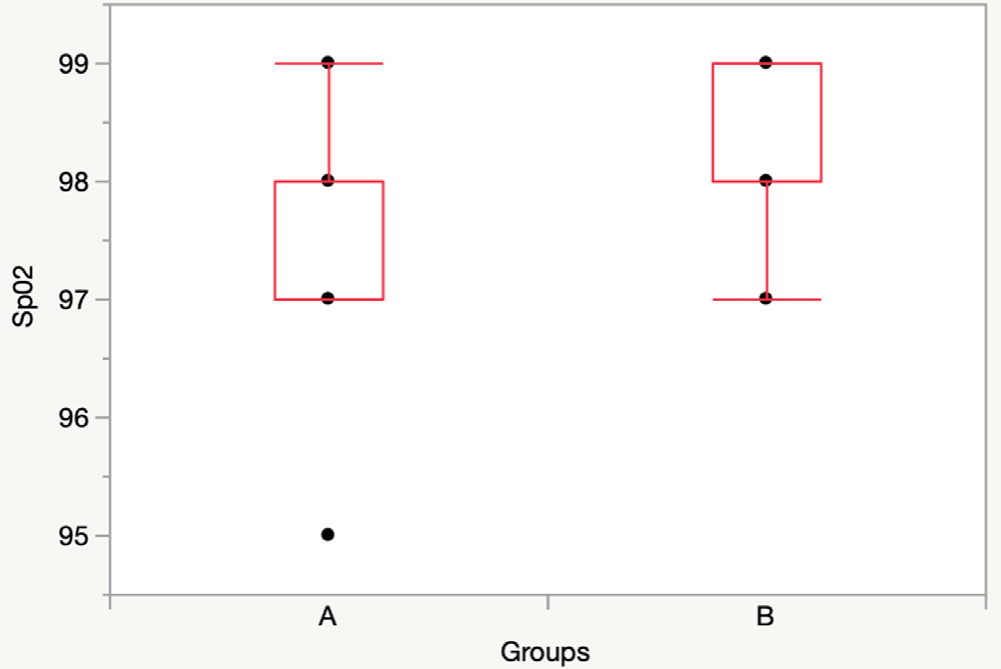
Comparison SpO2 between Group A (11 children) and Group B (11 children) at T30 (without mask).

**Fig. 2:**
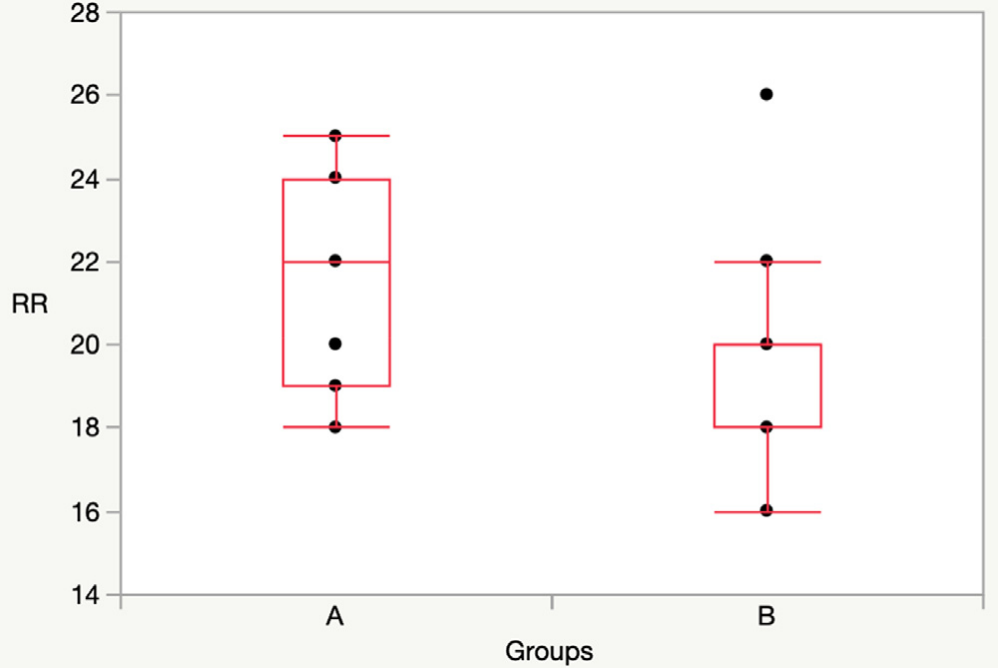
Comparison RR between Group A (11 children) and Group B (11 children) at T30 (without mask).

T30: Group A vs Group B: [SpO2: 98 (97-98) vs 98 (98-99), *p* = 0.22; RR: 22 (19-25) vs 20 (18-20), *p* = 0.12] (Figs. 1, 2).

At T60, when children wearing the mask, there was a significative difference in SpO2, while there was not difference in RR between Group A and Group B.

**Fig. 3:**
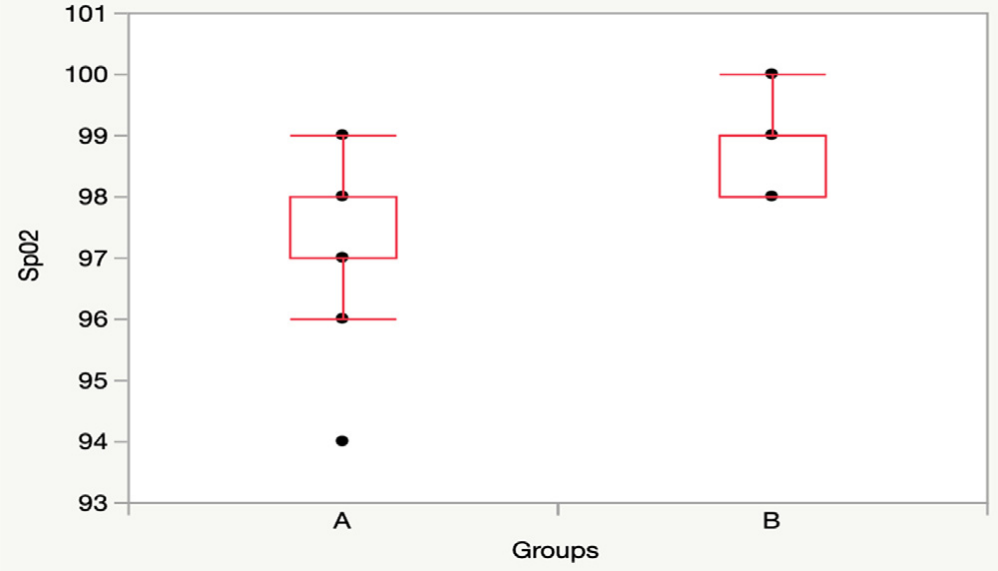
Comparison SpO2 between Group A (11 children) and Group B (11 children) at T60.

**Fig. 4:**
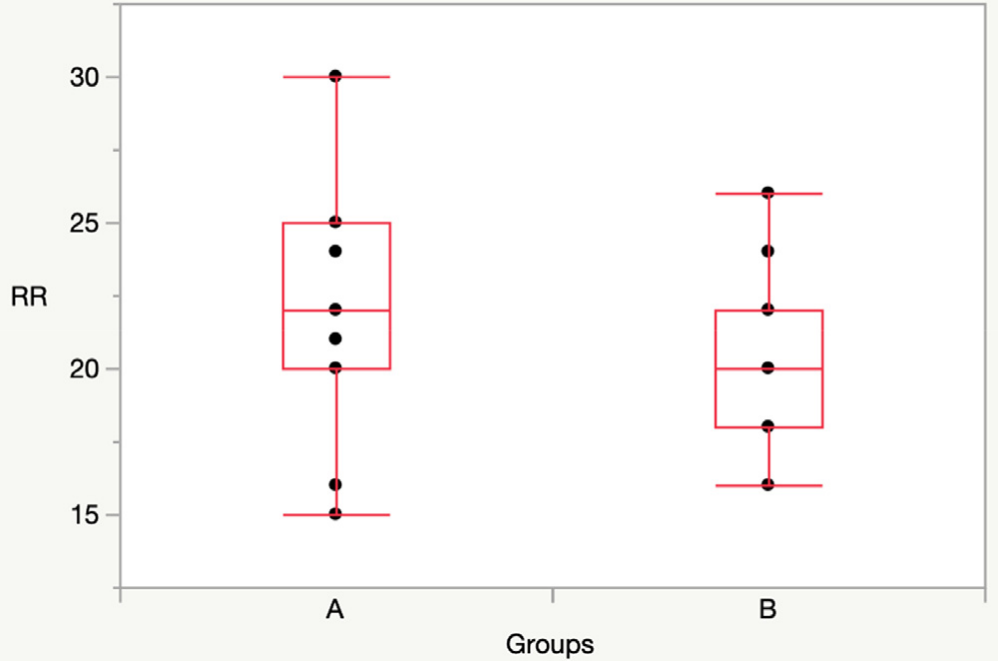
Comparison RR between Group A and Group B at T60.

T60: Group A vs Group B: [SpO2: 97 (97-98) vs 99 (98-99), *p* = 0.01]; RR: 22 (20-25) vs 20 (18-22), *p* = 0.22] (Figs. 3, 4).

At Twt, there was a significative difference in SpO2, while there was not difference in RR between Group A and Group B.

**Fig. 5:**
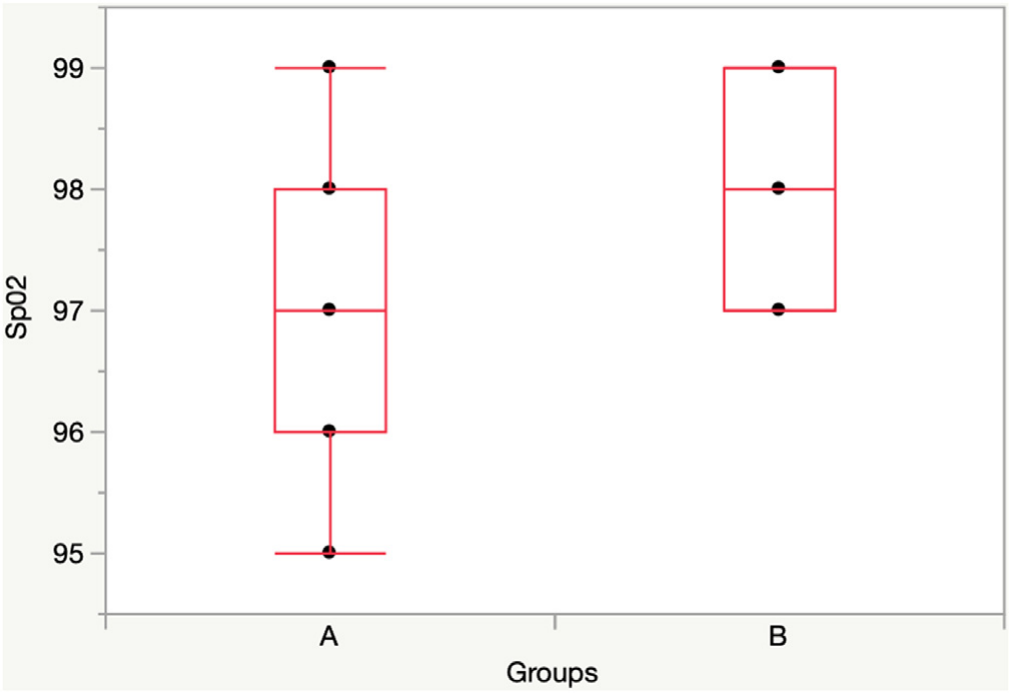
Comparison SpO2 between Group A and Group B at Twt.

**Fig. 6:**
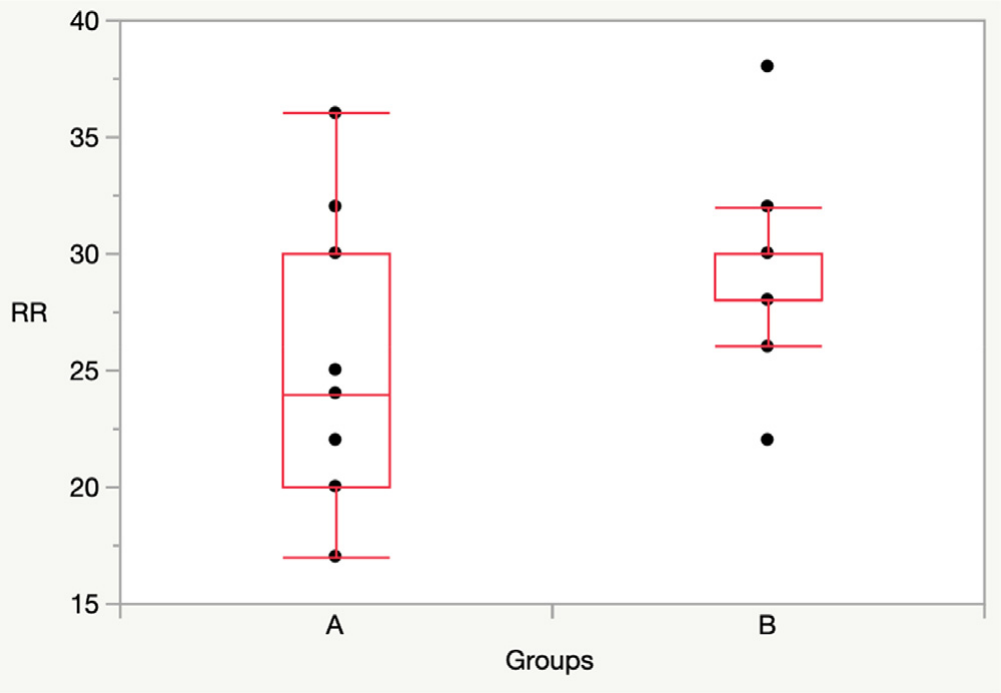
Comparison RR between Group A and Group B at Twt.

Twt: Group A vs Group B: [SpO2: 97 (96-98) vs 97 (98-99), *p* = 0.04]; RR: 24 (20-30) vs 28 (28-30), *p* = 0.059] (Figs. 5, 6).

The supplementary file contains the parameters monitored (oxygen saturation and respiratory rate) at each time (T30, T60, TWT) during the test for each of the two groups.

## 2 Experimental Design, Materials and Methods

This work was designed to test respiratory function in children wearing N95 masks. The study was conducted at Pediatric Unit of Santa Maria Goretti Hospital, Latina - Sapienza University of Rome from January 2021 to February 2021. The doctors of the Pediatric Unit of Santa Maria Goretti Hospital enrolled 25 healthy children without comorbidities and were not taking medications that influenced respiratory function. The aims were to test whether the use of the use of N95 mask without or with an exhalation valve in children was associated with episodes of desaturation or respiratory distress or with physical signs of respiratory distress.

3 children (younger than 3 years) refused to wear the mask and were excluded from the study.

The remaining 22 participants were randomly divided into two groups: Group A was composed of 11 children, 43% males and 57% females, the median age 96 months (72–108) that wearing N95 mask without an exhalation valve and Group B was composed of 11 children, 64% males and 36% females, the median age 96 months (72–108), that wearing N95 mask with an exhalation valve.

For every child enrolled, we implemented the following steps:

• Two days before the study started, a pediatrician examined the child to verify the individual's state of wellness and used Fit checking test (user seal-check, self-check) to verify good facial seal by the absence of air leaks using both positive and negative pressure checks; in the case of test failure, we modified the mask, tucking the edges into the inner surface, sewing them and moving the ear loops.
• The day of the test we performed three sessions: the first 30 min without a mask, the second 30 min while wearing the mask, and a third session consisting of a 12 min walking test, along a 40 m long corridor, while wearing the mask. In the second session children were encouraged to engage in their usual play activity, while during the walking test they were encouraged by the supervising doctor to walk fast, and the distance traveled was recorded.

 During the study every child was connected to a Masimo Patient Monitoring System (Rad-97™ with NomoLine Capnography) through nasocannulas and with saturation sensor to log oxygen saturation (%), respiratory rate (breaths/min), pressure of end-tidal carbon dioxide (mmHg), pulse rate (pulsation/min). All parameters were recorded every 15 min starting at 15 (T15), 30 (T30), 45 (T45), and 60 min (T60), and a final assessment at the end of the 12 min walk test (Twt). Two supervising physicians were assigned to record parameters during the test and to assess clinical signs of respiratory distress.

• Then, two doctors were assigned to deposit all collected data on an electronic database.

 Then, we performed statistical analysis: we used the Wilcoxon test to analyze the differences between the parameters recorded during the study in group A and B. A *p* < .05 was considered significant.

Data analysis was performed using JMP14.3.0 program for Mac by SAS Institute inc.

For the data expressed as continuous variables the approximation to normal of the distribution of the population was tested with the Shapiro-Wilk and Anderson Darling test. As results were asymmetrically distributed, data are expressed as median and interquartile range (IQR), 25th and 75th quartile, and non-parametric tests were used.  We used the Wilcoxon test to compare differences between the two groups at the five observation times.  A *p* < .05 was considered significant.

## Ethics Statement

This work was approved by Institutional review board of Maternal and Child Health Department of Latina Local Health Authority (protocol 01-09/03/21).

## CRediT Author Statement

**Riccardo Lubrano** Conceptualization Data curation Supervision; **Silvia Bloise** Data curation Writing – original draft Software Writing – review & editing; **Alessia Marcellino** Data curation Writing – original draft Software; **Claudia Proietti Ciolli** Visualization, Investigation; **Alessia Testa** Writing – review & editing; **Enrica De Luca** Data curation Writing – original draft Software; **Anna Dilillo** Visualization, Investigation; **Saverio Mallardo** Visualization, Investigation; **Sara Isoldi** Writing – review & editing; **Vanessa Martucci** Data curation Writing – original draft Software; **Mariateresa Sanseviero** Data curation Writing – original draft Software; **Emanuela Del Giudice** Writing – review & editing; **Concetta Malvaso; Claudio Iacovelli** Supervision; **Rita Leone** Supervision; **Donatella Iorfida** Visualization, Investigation; **Flavia Ventriglia** Supervision.

## Declaration of Competing Interest

The authors declare that they have no known competing financial interests or personal relationships which have or could be perceived to have influenced the work reported in this article.

## Appendix Supplementary materials

Image, application 1
